# Relationship Between Facial Types and Alveolar Crest Cortical Bone Thickness and CT Values Determined by Multidetector Computed Tomography

**DOI:** 10.3390/dj13090437

**Published:** 2025-09-22

**Authors:** Masahiro Kitano, Shin Ota, Shigeki Iijima, Ichiro Ogura

**Affiliations:** 1 Department of Oral and Maxillofacial Growth and Development, Orthodontics and Dentofacial Orthopedics, The Nippon Dental University Graduate School of Life Dentistry at Niigata, Niigata 951-8580, Japan; shige@ngt.ndu.ac.jp; 2 Department of Orthodontics, The Nippon Dental University School of Life Dentistry at Niigata, Niigata 951-8580, Japan; shin@ngt.ndu.ac.jp; 3 Department of Oral and Maxillofacial Radiology, The Nippon Dental University School of Life Dentistry at Niigata, Niigata 951-8580, Japan; ogura@ngt.ndu.ac.jp

**Keywords:** alveolar crest cortical bone thickness, computed tomography, facial type, multidetector CT

## Abstract

**Background/Objectives**: This study aimed to investigate the relationships between facial morphology and alveolar crest cortical bone thickness and to determine the computed tomography (CT) values using multidetector CT (MDCT). **Methods**: A total of 39 subjects were categorized into three groups based on the Frankfort mandibular plane angle: low angle, average angle, and high angle. The thickness of the alveolar crest cortical bone and CT values between the canines and first premolars and between the second premolars and first molars in the maxilla and mandible were measured and analyzed from pre-treatment MDCT images. The Kruskal–Wallis and Dunn–Bonferroni tests were applied to investigate the relationships between facial types and alveolar crest cortical bone thickness, and to determine the CT values. **Results**: Significant differences in cortical bone thickness between the mandibular premolar and first molar were observed when comparing the high-angle group with the low-angle group (*p* = 0.001) and the average-angle group with the low-angle group (*p* = 0.022). **Conclusions**: These findings indicate that examining facial type may reveal differences in anchor loss in the mandibular molar region, which could prove useful in formulating treatment plans.

## 1. Introduction

Various types of anchorages have been used in orthodontic treatment. However, none of the available intraoral appliances provide adequate anchorage, and extraoral appliances are unreliable without patient cooperation [1,2,3,4,5,6,7,8].

Skeletal anchorage methods, such as miniplates [1], mini-screw implants [2], and micro-screw implants [3], have been developed to address anchorage loss and are now widely adopted in clinical practice. Micro-screw implants offer several advantages, including ease of placement and removal, immediate loading, minimal anatomical restriction due to their small size, and lower cost compared to other skeletal anchorage methods. However, despite high success rates, they are not always available and may fail, making them difficult to use [4]. Controlling anchorage is important because anchorage loss may result in unsatisfactory orthodontic treatment results.

The morphology of the maxillomandibular complex, which is closely related to the surrounding musculature, is classified into three types: anteroposterior (sagittal), vertical, and transverse [5]. Vertical facial morphology is important for the orthodontist because it influences growth projections, anchorage systems, bite forces, and functions; additionally, it impacts orthodontic treatment goals and planning. The vertical dimension of the face is closely related to morphological changes influenced by genetics and childhood oral respiratory dysfunctions, which affect the formation of associated bone [6]. Unsurprisingly, patients with varying facial heights have different cortical bone thicknesses, as observed through multidetector computed tomography (MDCT) [7]. Moreover, crucial differences between MDCT and cone-beam CT (CBCT) complicate the use of quantitative gray values in the latter. Since the voxel intensity values of CBCT are arbitrary, Hounsfield unit (HU) values cannot be obtained correctly, and bone density cannot be evaluated [9].

To date, studies on implant sites have measured the density of the alveolar and basal bones on the buccal, palatal, and lingual sides of the maxilla and mandible [10,11]. We thought that if we could predict the ease of tooth movement by examining the alveolar crest cortical bone through which the teeth pass, we would be able to predict the level of anchorage required for each case, which would aid in orthodontic diagnosis. However, to the best of our knowledge, few studies measure the thickness and CT values of interdental alveolar crest cortical bone. This study aimed to investigate the relationships between facial morphology and alveolar crest cortical bone thickness, as well as CT values, using MDCT.

## 2. Materials and Methods

This study was approved by the ethics committee of the Nippon Dental University School of Life Dentistry at Niigata (approval no. ECNG-R-544, 11 October 2024) and was conducted in accordance with the Declaration of Helsinki. Written informed consent from the participants was waived in view of the retrospective use of medical records.

### 2.1. Patients

A total of 221 Japanese patients (165 females and 56 males) were retrospectively selected from the files of all orthodontic patients who had visited Nippon Dental University Niigata Hospital from September 2013 to September 2023. Patient selection was based on the following criteria: (1) availability of lateral cephalograms, panoramic radiographs, and CT imaging; (2) fully erupted maxillary and mandibular permanent teeth up to the second molars; (3) no prior orthodontic treatment; (4) absence of congenital diseases; and (5) no previous extraction of any permanent tooth.

The patients were classified into three facial type groups according to the Frankfurt mandibular plane angle (FMA): low-angle (<20.6° in females and <18.3° in males; n = 13), average-angle (20.7–31.5° in females and 18.4–26.2° in males; n = 104), and high-angle (>31.6° in females and >26.3° in males; n = 104). The categories for these groups were based on the Japanese FMA criteria (26.1 ± 5.4° for females and 22.3 ± 3.9° for males) [12]. To match the sample size, 13 patients were randomly selected from the average- and high-angle groups using the statistical package IBM SPSS Statistics, version 27 (IBM Japan, Tokyo, Japan). Thus, the total sample size in this study was 39, consisting of 27 females (age, 20.6 ± 6.2 years) and 12 males (age, 21 ± 4.8 years). All patient data were taken before orthodontic treatment.

### 2.2. Cephalometric Analysis

Lateral cephalograms were acquired using the same equipment (CX-150 SK, Asahi Roentgen, Kyoto, Japan) with standard settings; the images were used to assess FMA. After calibration, all cephalograms were traced and measured by one investigator (M.K.). Four reference points and two reference lines were established. One angular measurement was made on tracing paper using a protractor with an accuracy of 0.5 degrees.

An investigator (M.K.) performed the first FMA measurement, and 19 lateral cephalograms were randomly selected for a second measurement 3 months later. A paired *t*-test revealed no significant errors between the first and second measurements (*p* > 0.5). Random errors, evaluated using the Dahlberg formula [13], were less than 0.3°.

### 2.3. CT Imaging Analysis

CT imaging was performed using a 16-MDCT scanner (Aquilion TSX-101A; Canon Medical Systems, Otawara, Japan) with a protocol routinely employed for orthodontics in our hospital: tube voltage, 120 kV; tube current, 150 mA; field of view, 240 × 240 mm; and rotation time, 0.5 s. A single investigator (M.K.) measured all the CT images.

**Figure 1:**
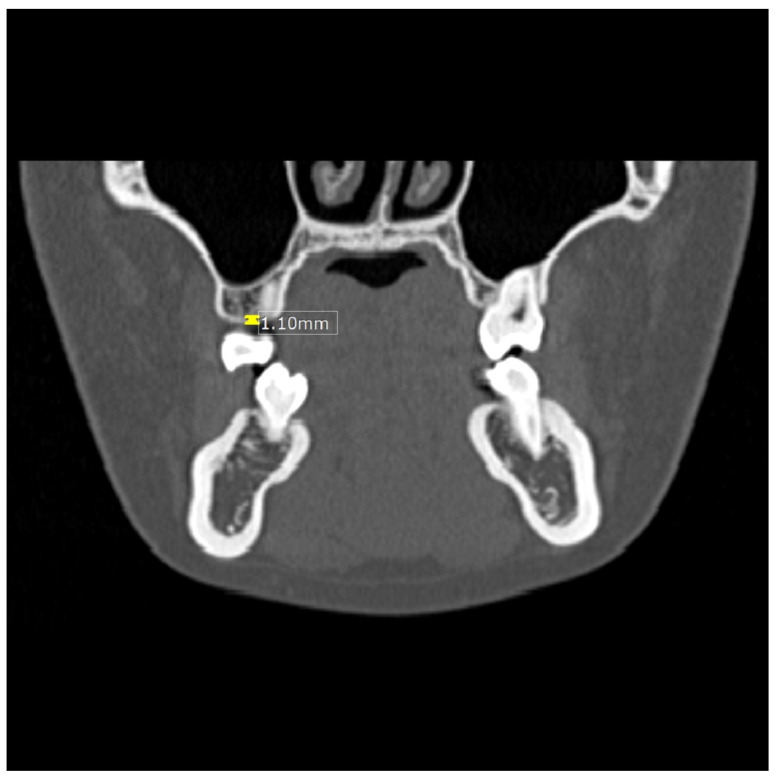
Image showing measurement of the thickness (mm) of the alveolar crest cortical bone in the maxilla.

**Figure 2:**
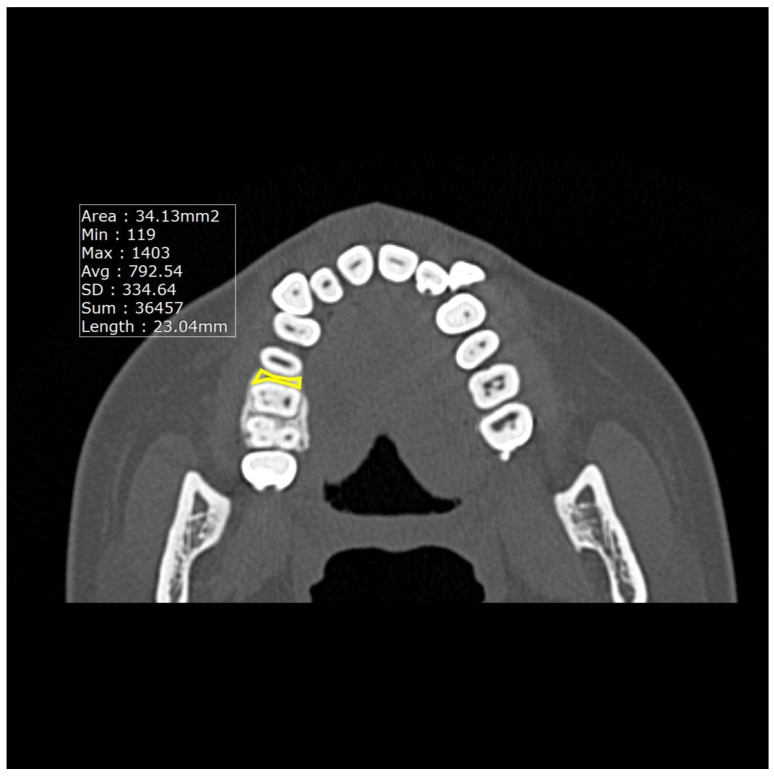
Image showing the measurement of the average CT values in the maxilla.

Measurements of the thickness of the alveolar crest cortical bone (Figure 1) were taken in the coronal plane from four sites: thickness between the maxillary canine and first premolar (Tmax3), thickness between the mandibular canine and first premolar (Tman3), thickness between the maxillary premolar and first molar (Tmax6), and thickness between the mandibular premolar and first molar (Tman6). Likewise, the densities of the CT values (Figure 2) between the maxillary canine and first premolar (Dmax3), the mandibular canine and first premolar (Dman3), the maxillary premolar and first molar (Dmax6), and the mandibular premolar and first molar (Dman6) were also obtained in the axial plane.

The average CT values were measured using a CT workstation and software (INFINITT JAPAN, Tokyo, Japan). Previous studies have reported that the cortical bone width is the same on both sides [14], so some have measured it on one side only [10,11]. Therefore, in this study, only one side was measured. Cortical bone widths were measured three times, with the results comprising the average of the three measurements.

### 2.4. Statistical Analysis

Statistical analyses were performed using IBM SPSS Statistics, version 27 (IBM Japan, Tokyo, Japan). The means and standard deviations were calculated for each measurement in each group. The Kruskal–Wallis test was performed to analyze the effect of facial types on alveolar crest cortical bone thickness and CT values, and the Dunn–Bonferroni test was performed for multiple comparisons. The level of statistical significance for all analyses was set at *p* < 0.05.

## 3. Results

Table 1 shows the results for all samples and all measurement sites.

Table 2 shows the alveolar crest thickness and CT values between premolar and first molar of the maxillary and mandibular. The results are shown in order of highest value for each measurement site: Tmax6: average, 1.30 ± 0.18 mm; low, 1.22 ± 0.32 mm; high, 1.10 ± 0.26 mm; Tman6: low, 1.66 ± 0.78 mm; average, 1.14 ± 0.25 mm; high, 1.03 ± 0.20 mm; Dmax6: average, 1016.16 ± 190.28; low, 902.36 ± 152.10; high, 889.68 ± 176.91; and Dman6: average, 997.29 ± 261.70; low, 953.16 ± 236.38; high, 885.38 ± 241.38.

Table 3 shows the alveolar crest thickness and CT values between the canine and first premolar of the maxilla and mandible. The results are shown in order of highest value for each measurement site: Tmax3: low, 1.34 ± 0.31 mm; average, 1.29 ± 0.31 mm; high, 1.15 ± 0.17 mm; Tman3: low, 1.34 ± 0.26 mm; average, 1.32 ± 0.23 mm; high, 1.26 ± 0.15 mm; Dmax3: low, 1048.25 ± 196.13; average, 983.10 ± 98.71; high, 970.30 ± 94.77; and Dman3: high, 1110.42 ± 155.73; low, 1072.96 ± 255.48; average, 1055.33 ± 162.38.

The Kruskal–Wallis test revealed statistically significant (*p* < 0.05) differences in Tman6 (Table 2) and Dmax3 (Table 3) among the three groups. The post hoc test showed significant differences in Tman6 between the high- and low-angle groups (*p* = 0.001 *) and the average- and low-angle groups (*p* = 0.022 *). In the case of Dmax3, the difference between the high-angle and low-angle groups approached statistical significance (*p* = 0.068). No statistically significant differences were observed at the other measurement sites.

## 4. Discussion

According to Pauwels et al. [15], bone quality parameters and classifications are primarily based on bone density, which can be estimated using Hounsfield units (HU) derived from MDCT datasets. CT images in DICOM format contain data in CT values, allowing the INFINITT PACS version 3.0.11.5 software program to measure them. Maki et al. [16] reported a high correlation between average CT values and hydroxyapatite concentrations in a study using fresh cadavers. Norton and Gamble [17] stated that using CT scans to measure HU offers an accurate way to assess bone density. Since MDCT can obtain the correct CT value for bone, the evaluation of bone density with MDCT can be more accurate than with CBCT. Based on these findings, we used MDCT scans to measure cortical bone thickness and density in the current study.

Masumoto et al. [18] and Tsunori et al. [7] examined three-dimensional (3D) images of dry Asian skulls and found a correlation between facial types and mandibular cortical bone thickness; the lingual cortical bone thickness in molar sections from the short-faced group was greater than that in sections from the average- and long-faced groups. According to Ozdemir et al. [11], the thickness of the cortical bone in the jaw is closely related to vertical facial types; in their study, patients with low-angle faces exhibited significantly greater cortical bone thickness than those with high-angle faces at all measurement sites (mandibular buccal, maxillary buccal, and maxillary palatal alveolar bones). Park et al. [10] reported that cortical bone density increases from the incisor to the molar region in the maxilla and mandible.

In the current study, the alveolar crest cortical bone in the mandibular molar region was significantly thicker in the low-angle group than in the high- and average-angle groups. No significant differences in alveolar crest cortical bone thickness were observed in the maxillary canine, maxillary molar, and mandibular canine regions among the three groups. Likewise, no significant differences in CT values were observed at any of the measurement sites among the three groups.

These results were consistent with those of previous studies on the thickness of mandibular cortical bone [7,11,18]; however, they differed from those on the thickness of the maxillary cortical bone [11]. In addition, a recent report using quantitative CT showed no significant difference in bone mineral density in the maxillary interradicular sites from the canines to the second molars. In the mandible, there was a significant difference between the interradicular bone mineral density, and a tendency to increase from the anterior to the posterior region was observed [19]. These discrepancies may be attributed to differences in the measurement sites.

The rate of tooth movement appears to be related to bone density. Experiments in animals with induced bone mineral density loss and altered bone metabolism following nutritional hyperthyroidism [20] and acute and chronic corticosteroid treatment [21] have shown rapid tooth movement. The rate of tooth movement in cortical bone was slower than in cancellous bone [22]. The findings of the current study indicate that anchor loss of mandibular molars is less likely to occur at low angles.

Our study compared vertical facial types using FMA. A study investigating mandibular bone thickness from the anteroposterior skeletal relationships using the ANB angle reported that as mandibular protrusion increased, the thickness of the mandibular ramus decreased, but the thickness of the mandibular body remained unchanged [23]. Anteroposterior skeletal relationships using the ANB angle may also affect alveolar crest thickness.

A scoping review of the success–failure rates of temporary anchorage devices in orthodontics reported that the success rates of temporary anchorage devices were over 90% [24]. Although these have high success rates, temporary anchorage devices may fail, so adequate diagnostics should be performed.

The primary limitation of this study was the relatively small sample size, which did not allow for comparisons by sex or age. Thus, future studies with larger samples are needed to confirm moderate or small differences. Furthermore, by investigating differences based on sex and age, and by increasing the number of measurement sites and investigating differences between sites, it may be possible to predict the level of difficulty of a case if the patient’s age, sex, and facial type are known.

## 5. Conclusions

The cortical bone in the mandibular molar area was significantly thicker in the low-angle group in this study, which could reduce the likelihood of anchor loss in the mandibular molar. Moreover, our findings suggest that distinguishing between facial types may be beneficial in developing a more effective treatment plan. In high-angle and average-angle cases, skeletal anchorage methods and extraoral appliances in the maxillary and mandibular regions should be considered to prevent loss of anchorage [25]. Conversely, excessive attention to anchorage loss may not be necessary in low-angle cases. However, when large mesial movement of the mandibular molars is planned, the use of skeletal anchorage methods should be considered, regardless of the case type [26].

## Figures and Tables

**Table 1:** The alveolar crest thickness and CT values of the maxillary and mandibular (n = 39).

Sample	Sex	Age	FMA	Tmax6	Dmax6	Tman6	Dman6	Tmax3	Dmax3	Tmax3	Dman3
1	Female	32	10.0	1.01	971.76	4.06	1384.63	1.29	1231.00	1.24	1155.78
2	Male	33	10.5	1.16	982.15	1.79	1067.52	1.72	912.97	1.31	769.87
3	Female	19	11.5	0.98	625.90	1.18	724.50	1.18	1187.47	1.18	1148.24
4	Female	22	12.0	1.07	814.62	1.15	1116.06	2.08	1038.58	1.68	1262.38
5	Female	19	15.0	2.00	1114.10	1.73	957.52	1.33	1123.86	1.47	1288.96
6	Female	22	15.0	0.98	1097.40	2.00	1070.07	1.31	1168.95	1.55	1213.24
7	Male	18	16.0	0.77	791.43	1.56	699.93	1.02	492.52	1.03	529.06
8	Male	19	16.5	1.47	840.63	1.08	680.16	1.33	1073.26	1.58	1185.36
9	Male	25	17.0	1.30	772.38	1.43	735.75	0.81	1061.57	1.25	966.00
10	Female	23	17.0	1.34	896.17	1.62	1072.45	1.41	1186.56	1.22	1323.65
11	Female	16	17.0	1.02	766.59	1.53	815.00	1.46	1099.08	1.96	1022.00
12	Female	20	18.0	1.20	1118.21	1.36	1304.42	1.29	1163.09	1.39	1349.67
13	Female	13	18.0	1.60	939.29	1.10	763.08	1.22	888.29	1.12	734.21
14	Male	20	18.0	1.29	992.75	1.15	869.88	1.41	1017.67	1.88	1041.89
15	Male	16	19.0	1.21	889.43	0.87	608.10	1.35	926.73	1.24	849.50
16	Female	18	21.0	1.28	1385.71	1.79	1313.37	1.22	1108.55	1.46	1405.83
17	Female	19	21.0	1.24	1091.46	0.98	1146.95	1.09	1021.50	1.00	1179.28
18	Male	21	21.5	1.44	1018.85	1.12	897.22	1.01	1153.06	1.13	1157.10
19	Female	21	22.5	1.64	850.85	0.98	906.15	1.34	896.45	1.20	919.39
20	Female	24	24.0	1.03	1022.15	1.03	833.80	1.35	944.16	1.28	1087.81
21	Female	22	24.5	1.25	988.40	1.22	1194.54	1.34	1004.67	1.36	1029.54
22	Female	15	26.5	1.06	899.64	1.14	580.15	0.89	801.67	1.11	874.00
23	Female	44	26.5	1.41	1100.33	1.41	1495.83	1.41	847.64	1.16	1127.33
24	Female	12	26.5	1.11	623.47	1.26	972.31	1.00	1040.83	1.38	955.00
25	Male	22	26.5	1.16	1215.11	1.48	1293.94	1.52	1057.77	1.10	1179.88
26	Male	19	27.0	0.94	806.34	1.10	1070.22	1.12	1108.55	1.47	1015.52
27	Female	24	28.0	1.46	1066.00	1.04	992.13	2.14	985.71	1.40	877.76
28	Male	14	28.0	1.39	828.71	0.93	779.42	1.25	1072.93	1.10	998.67
29	Male	23	28.0	1.10	512.27	0.74	744.91	1.12	901.33	1.23	1042.15
30	Female	20	29.0	1.51	1281.00	0.84	1154.29	1.25	1031.63	1.59	1214.85
31	Male	22	31.5	0.96	798.13	1.15	980.12	1.03	910.09	1.34	982.29
32	Female	20	32.0	1.48	1088.23	1.03	1081.63	1.19	1053.59	1.15	1037.00
33	Female	18	32.5	1.22	803.04	1.09	425.12	0.99	786.60	1.25	1072.25
34	Female	13	32.5	1.07	919.09	0.89	643.00	1.19	839.79	1.56	924.44
35	Female	22	33.0	1.49	856.38	1.15	1048.76	1.27	980.37	1.23	1000.20
36	Female	18	33.0	1.05	841.43	1.20	677.97	0.95	982.00	1.14	1452.00
37	Female	19	34.5	1.03	877.65	0.91	732.68	0.86	1025.44	1.44	1128.17
38	Female	20	35.0	0.75	891.42	0.75	911.53	1.21	941.07	1.29	1307.00
39	Female	22	45.5	0.64	1128.00	1.02	1120.67	1.28	954.35	1.10	1295.86

**Table 2:** Comparison of the alveolar crest thickness and computed tomography (CT) values between the second premolar and first molar regions across the three facial types.

	High Angle (n = 13)	Average Angle (n = 13)	Low Angle (n = 13)	*p* Value
Alveolar crest thickness (mm)				
Tmax6 ± SD	1.10 ± 0.26	1.30 ± 0.18	1.22 ± 0.32	0.102
Tman6 ± SD	1.03 ± 0.20	1.14 ± 0.25	1.66 ± 0.78	0.001 *
CT value (HU)				
Dmax6 ± SD	889.68 ± 176.91	1016.16 ± 190.28	902.36 ± 152.10	0.120
Dman6 ± SD	885.38 ± 241.38	997.29 ± 261.70	953.16 ± 236.38	0.587

Values are presented as mean ± standard deviation (SD). * *p* < 0.05; Tmax6, thickness of between the maxillary premolar and first molar; Tman6, thickness of between the mandibular premolar and first molar; Dmax6, density of between the maxillary premolar and first molar; Dman6, density of between the mandibular premolar and first molar; HU, Hounsfield unit. Dunn–Bonferroni test for L6W: High angle—Average angle (*p* = 1.000), High angle—Low angle (*p* = 0.001 *), Average angle—Low angle (*p* = 0.022 *).

**Table 3:** Comparison of the alveolar crest thickness and computed tomography (CT) values between the canine and first premolar regions across the three facial types.

	High Angle (n = 13)	Average Angle (n = 13)	Low Angle (n = 13)	*p* Value
Alveolar crest thickness (mm)				
Tmax3 ± SD	1.15 ± 0.17	1.29 ± 0.31	1.34 ± 0.31	0.085
Tman3 ± SD	1.26 ± 0.15	1.32 ± 0.23	1.38 ± 0.26	0.430
CT value (HU)				
Dmax3 ± SD	970.30 ± 94.77	983.10 ± 98.71	1048.25 ± 196.13	0.043 *
Dman3 ± SD	1110.42 ± 155.73	1055.33 ± 162.38	1072.96 ± 255.48	0.669

Values are presented as mean ± standard deviation (SD). * *p* < 0.05. Tmax3, thickness between the maxillary canine and first premolar; Tman3, thickness between the mandibular canine and first premolar; Dmax3, density between the maxillary canine and first premolar; Dman3, density between the mandibular canine and first premolar; HU, Hounsfield unit. Dunn–Bonferroni test for U3D: High angle—Average angle (*p* = 1.000), High angle—Low angle (*p* = 0.068), Average angle—Low angle (*p* = 0.119).

## Data Availability

The original contributions presented in this study are included in the article. Further inquiries can be directed to the corresponding author.

## Abbreviations

The following abbreviations are used in this manuscript:

CT	computed tomography
MDCT	multidetector computed tomography
CBCT	cone-beam computed tomography
FMA	Frankfurt mandibular angle
Tmax3	thickness between the maxillary canine and first premolar
Tman3	thickness between the mandibular canine and first premolar
Tmax6	thickness between the maxillary premolar and first molar
Tman6	thickness between the mandibular premolar and first molar
Dmax3	density between the maxillary canine and first premolar
Dman3	density between the mandibular canine and first premolar
Dmax6	density between the maxillary premolar and the first molar
Dman6	density between the mandibular premolar and the first molar
SD	standard deviation
HU	Hounsfield unit
